# Validity and reliability of the Chinese beliefs about medicines questionnaire–specific in caregivers of children with Epilepsy

**DOI:** 10.3389/fpubh.2025.1603132

**Published:** 2025-12-19

**Authors:** Xixi Jiang, Qiuji Tao, Yawen Xue, Chunsong Yang, Rong Luo

**Affiliations:** 1 Nursing Unit of Pediatric Neurology, West China Second University Hospital, Sichuan University, Chengdu, China; 2 Key Laboratory of Birth Defects and Related Diseases of Women and Children (Sichuan University), Ministry of Education, Chengdu, China; 3 Department of Pharmacy, Evidence-based Pharmacy Center, West China Second Hospital, Sichuan University, Chengdu, China; 4 Department of Pediatrics, West China Second University Hospital, Sichuan University, Chengdu, China

**Keywords:** epilepsy, beliefs about medicine–specific, validity, reliability, caregivers, children

## Abstract

**Introduction:**

There are 10.5 million children with epilepsy (CWE) around the world. Approximately 70% of people with epilepsy could become seizure-free with appropriate antiseizure therapy. Beliefs may play an important role in medication adherence according to the Health Belief Model. The Belief about Medicines Questionnaire–Specific (BMQ-S) was developed to assess individuals’ beliefs about medicines, yet few studies have examined its application among caregivers of CWE in China.

**Objective:**

The study aimed to verify the validity and reliability of the Chinese BMQ-S among caregivers of CWE.

**Materials and methods:**

A cross-sectional study was conducted in West China Second University Hospital, Sichuan University, from June 2021 to May 2024. The Chinese version of the BMQ-S, originally validated for depression, was adapted for use in this study. After obtaining informed consent, participants were asked to complete a general information questionnaire and the Chinese BMQ-S. Reliability was assessed using McDonald’s omega in SPSS 26 (IBM Corporation, Armonk, NY, USA), and construct validity was evaluated using confirmatory factor analysis in Mplus 8.1 (Muthén & Muthén, Los Angeles, CA, USA). The Morisky Medication Adherence Scale was used to assess medication adherence of CWE. The relationship between BMQ-S and adherence was explored using the binary logistic regression analysis.

**Results:**

A total of 2,730 caregivers were recruited, of whom 2,405 (88.01%) completed the survey. The children of participants included 1,283 (53.35%) boys and 1,122 (46.65%) girls, ranging in age from 0.08 to 17.80 years. McDonald’s omega values were 0.808 for BMQ-necessity and 0.709 for BMQ-concern. Confirmatory factor analysis showed the following fit indices for the final two-factor model: comparative fit index = 0.975, Tucker–Lewis index = 0.964, standardized root mean square residual = 0.038, and root mean square error of approximation = 0.079. The results indicated that 1,513 CWE (62.91%) were adherent to their medication therapy and 892 (37.09%) were non-adherent. However, no statistically significant association was observed between BMQ-S scores and medication adherence.

**Conclusion:**

The Chinese BMQ-S is a reliable and valid tool for assessing medicine beliefs among caregivers of CWE. Further studies are needed to explore the relationship between BMQ scores and antiseizure medicine adherence.

## 1 Introduction

Epilepsy is a brain disorder characterized by a persistent predisposition to generate recurrent seizures (1). Worldwide, approximately 50 million people are affected by epilepsy, with children accounting for 25% of the cases (2, 3). In China, it is estimated that approximately 10 million individuals have epilepsy. Although the prevalence of epilepsy among children varies across different studies, it is expected to increase (4, 5). Children with epilepsy (CWE) face increased risks of certain comorbidities, including attention-deficit/hyperactivity disorder (6), autism spectrum disorder (7), psychiatric disorders (8), and even an increased risk of mortality (9). These issues adversely affect children’s quality of life and increase the burden on their families.

Medicines are the cornerstone of epilepsy treatment. Approximately 70% of people suffering from epilepsy could become seizure-free with appropriate medication therapy (2). However, a recent study investigating the adherence to treatment in CWE in China found that only 21.3% of children exhibited good adherence, while 27.3% showed poor adherence. Non-adherence to therapy remains a common problem in children and sifnificantly reduces the likelihood of being seizure-free or achieving a cured state (10, 11).

Factors such as child’s age, family support, and parents’ marriage status are associated with adherence (12). The Health Belief Model (HBM) proposed by Rosenstock in 1974 posits that health-related beliefs play an important role in health-related behaviors. According to the HBM, health beliefs encompass perceived susceptibility to and the severity of an illness and perceived benefits and barriers to adopting health actions. These perceptions collectively determine health-related actions (13). Medicine adherence, as a health action involving taking medicines in the correct dosage, for the appropriate duration, and at the right times, can be improved by strengthening beliefs (14). A study conducted in China showed that parents’ perceptions of the severity of their child’s condition and their own self-efficacy positively influence their children’s adherence, whereas perceived susceptibility has a negative impact on adherence (15).

To assess people’s beliefs about medicines, the Belief about Medicines Questionnaire-Specific (BMQ-S) was developed. The BMQ-S is a clear and concise tool that could be used to evaluate the beliefs of caregivers of CWE regarding specific prescribed medications (i.e., antiseizure medicines), and it has been translated into multiple languages and applied to various chronic disease populations (16–20). Although the Chinese version of the BMQ-S has been validated in patients with depression, asthma, diabetes, and hypertension, few studies, to the best of our knowledge, have examined its application among CWE caregivers (21, 22). This study aimed to verify the reliability and validity of the Chinese BMQ-S in measuring beliefs about medicines among caregivers of CWE.

## 2 Materials and methods

### 2.1 Study design

A cross-sectional survey was conducted at West China Second University Hospital, Sichuan University, in Chengdu, China, between June 2021 and May 2024. Caregivers whose children met the following eligibility criteria were recruited:

1. Children older than 1 month and younger than 18 years old.
2. Children diagnosed with epilepsy in accordance with guidelines issued by the International League Against Epilepsy (1).
3. Children received antiseizure medicine treatment.
4. Both children and their caregivers agreed to participate in the survey.

Meanwhile, the study exclusion criteria were as follows:

1. Children’s caregivers who were unable to communicate in Chinese.
2. Caregivers with a medically confirmed mental disorder.
3. Absence of informed consent.

### 2.2 Measurements

#### 2.2.1 The general questionnaire

The general questionnaire was used to collect demographic information, including the child’s sex, age, weight, height, and residency, and their caregiver’s marital status, employment, education level, and monthly family income.

#### 2.2.2 BMQ-S

The BMQ-S consists of two subscales: necessity (BMQ-N) and concern (BMQ-C), with a total of 10 items scored on a 5-point Likert scale (1 = strongly disagree and 5 = strongly agree). The BMQ-N includes five items assessing the belief that the prescribed specific medicine is necessary to maintain health; higher scores reflect greater perceived necessity. The BMQ-C includes five items evaluating concerns about adverse or long-term effects, with higher scores indicating greater concern (20). The Chinese BMQ-S was initially translated by Yang et al. Their study demonstrated good reliability (Cronbach’s alpha: 0.813 for BMQ-N, 0.706 for BMQ-C) and validity (average content validity index: 0.96; 64.56% of the total variance explained by three factors) (23). The name of the specific medication was modified from “antidepressants” to “antiseizure medicines,” and caregivers were instructed to answer the items in reference to their child’s medicines (24). Example items include: “My child’s health, at present, depends on antiseizure medicines” (from the BMQ-N) and “I sometimes worry about the long-term effects of antiseizure medicines on my child” (from the BMQ-C).

To assess the association between beliefs about medications and adherence, four attitude groups were created based on the average scores of necessity and concern: (a) low necessity scores and low concern scores imply “Indifference,” (b) high necessity scores and low concern scores imply “Acceptance,” (c) low necessity scores and high concern scores imply “Skepticism,” and (d) high necessity scores and high concern scores imply “Ambivalence” (25). The necessity–concerns differential reflects a cost–benefit evaluation of children’s medicines. This differential is calculated by subtracting the concern score from the necessity score, producing a range from −20 to 20 (25, 26).

#### 2.2.3 The Morisky medication adherence scale-4 items (MMAS-4)

The MMAS-4 was used to evaluate the adherence among CWE. The MMAS-4 includes four items with yes/no responses, where a “no” answer is scored as 1 point, and a “yes” answer is scored as 0 points. The total score range is 0–4 points. A score of 4 points indicate adherence, whereas any score below 4 points indicates non-adherence. Despite its developer’s report of unideal reliability (Cronbach’s alpha = 0.61), likely due to the limited number of items, the scale demonstrated good concurrent and predictive validity (27). A license for the use of the MMAS-4 from MMAS Research LLC (Coronado, CA, USA) has been obtained.

### 2.3 Data collection

The child’s physician identified and recruited eligible caregivers of CWE at West China Second University Hospital, Sichuan University, during routine care sessions. After obtaining written informed consent, an SMS text message containing a URL link to the online questionnaire was sent to caregivers’ mobile phones through the hospital’s follow-up platform. Before participating, caregivers were informed about the study’s purpose, voluntary nature, and data confidentiality. To ensure data quality, researchers reviewed submissions, contacted participants to resolve missing data, and clarified ambiguous responses by phone. The study was approved by the ethics committee of West China Second University Hospital, Sichuan University.

### 2.4 Statistical analysis

The collected data were analyzed in SPSS 26 (IBM Corporation, Armonk, NY, USA) and expressed as mean or percentage values based on the distribution of data. Binary logistic regression analysis was performed to identify potential demographic factors associated with adherence and examine the relationship between BMQ-S scores and adherence.

#### 2.4.1 Reliability

Reliability reflected the consistency of a measure across repeated assessments. McDonald’s omega was applied to evaluate internal consistency reliability and was calculated by the OMEGA macro in SPSS 26 (28, 29). A value of at least 0.7 was considered acceptable (30).

#### 2.4.2 Validity

The average variance extracted (AVE) value was used to assess convergent validity, with values greater than 0.5 considered acceptable. Construct validity indicates the degree to which test results correspond to theoretical constructs (30). Given that the BMQ-S has been validated in various populations, we hypothesized good validity among CWE caregivers. Confirmatory factor analysis (CFA) was performed using the weighted least squares means and variance adjusted (WLSMV) method in Mplus 8.1. Model fit was assessed using the comparative fit index (CFI), Tucker–Lewis index (TLI), standardized root mean square residual (SRMR), and root mean square error of approximation (RMSEA). The cutoff of the fit indices was ≥0.90 for the CFI and TLI and ≤0.08 for SRMR and RMSEA, respectively.

## 3 Results

### 3.1 Participant characteristics

A total of 2,730 questionnaires were distributed, with 2,405 (88.10%) valid responses subsequently collected. Among the participants’ children, there were 1,283 boys (53.35%) and 1,122 girls (46.65%), with ages ranging from 0.03 to 17.80 years. Child’s age, height, weight, siblings, caregiver’s age, monthly income, marriage status, education level, and residency were identified as covariates associated with adherence (Table 1).

**Table 1:** Medication adherence according to participant characteristics.

Characteristics	*N* (%) /Mean ± SD	Adherence	OR	95% CI
Children				
Sex			1.06	(0.90, 1.25)
Boys	1,283 (53.35)	799 (62.28)		
Girls	1,122 (46.65)	714 (63.64)		
Age (years)***	8.25 ± 3.87	7.56 ± 3.71	0.88	(0.86, 0.90)
Height (cm) ***	120.94 ± 52.60	117.88 ± 62.93	0.99	(0.99, 1.00)
Weight (kg) ***	25.68 ± 13.51	23.74 ± 12.65	0.97	(0.97, 0.98)
Siblings***			0.67	(0.56, 0.79)
No	1,377 (57.26)	811 (76.87)		
Yes	1,028 (42.74)	702 (68.29)		
Caregivers				
Monthly income (yuan)*				
≤ 5,000	1,055 (43.87)	617 (58.48)	Ref	
5001–10,000	907 (37.71)	591 (65.16)	1.33	(1.11, 1.60)
≥ 10,000	443 (18.42)	305 (68.85)	1.57	(1.24, 1.99)
Age (years)				
≤ 30	381 (15.84)	254 (66.67)	Ref	
31–44	1764 (73.35)	1,110 (62.93)	0.85	(0.67, 1.07)
45–59*	237 (9.85)	137 (57.81)	0.69	(0.49, 0.96)
≥ 60	23 (0.96)	12 (52.17)	0.55	(0.23, 1.27)
Employment status			1.01	(0.85, 1.20)
Unemployed	951 (39.54)	597 (62.78)		
Employed	1,454 (60.46)	916 (63.00)		
Marriage status***			1.78	(1.32, 2.40)
Single	186 (7.73)	93 (50.00)		
Married	2,219 (92.27)	1,420 (63.99)		
Education level***				
High school or lower	1,242 (51.64)	708 (57.00)	Ref	
University	1,094 (45.49)	756 (69.10)	1.69	(1.42, 2.00)
Master’s degree or higher	69 (2.87)	49 (71.01)	1.85	(1.09, 3.15)
Residency***				
Rural	692 (28.77)	383 (55.35)	Ref	
Suburb	428 (17.80)	264 (61.68)	1.30	(1.02, 1.66)
Urban	1,285 (53.34)	866 (67.39)	1.67	(1.38, 2.02)
Family history of epilepsy				
No	2,246 (93.39)	1,408 (62.69)	1.16	(0.82, 1.63)
Yes	159 (6.61)	105 (66.04)		

Code: adherence: 0 = non-adherence, 1 = adherence; Ref, reference group; CI, confidence interval; OR, odds ratio; * *p* < 0.05, **p < 0.01, ***p < 0.001.

### 3.2 Reliability and validity

Scores for both the BMQ-N and BMQ-C ranged from 5 to 25 points. The mean score for the BMQ-N was 16.86 ± 3.23 points and that for the BNQ-C was 17.94 ± 3.02 points. McDonald’s omega values and AVE values for the BMQ-N and BMQ-C after adaptation were greater than 0.7 and 0.5, respectively. The detailed results are displayed in Table 2.

**Table 2:** BMQ-S item wording, mean scores, and reliability indices.

Item	Mean ± SD (points)	Model 1		Model 2^#^	
		AVE	McDonald’s omega	AVE	McDonald’s omega
Necessity		0.54	0.808	0.55	0.808
1. My health, at present, depends on my medicines	3.47 ± 0.71	✓		✓	
3. My life would be impossible without my medicines	2.92 ± 0.91	✓		✓	
4. Without my medicines, I would be very ill	3.26 ± 0.87	✓		✓	
7. My health in the future will depend on my medicines	3.47 ± 0.74	✓		✓	
10. My medicines protect me from becoming worse	3.76 ± 0.57	✓		✓	
Concern		0.39	0.734	0.54	0.709
2. Having to take medicines worries me	3.70 ± 0.66	✓		✓	
5. I sometimes worry about the long-term effects of my medicines	3.75 ± 0.63	✓		✓	
6. My medicines are a mystery to me	4.10 ± 0.51	✓			
8. My medicines disrupt my life	3.12 ± 0.06	✓		✓	
9. I sometimes worry about becoming too dependent on my medicines	3.27 ± 0.86	✓		✓	

^#^ Model 2 excluded item 6 from the BMQ-C subscale.

**Figure 1:**
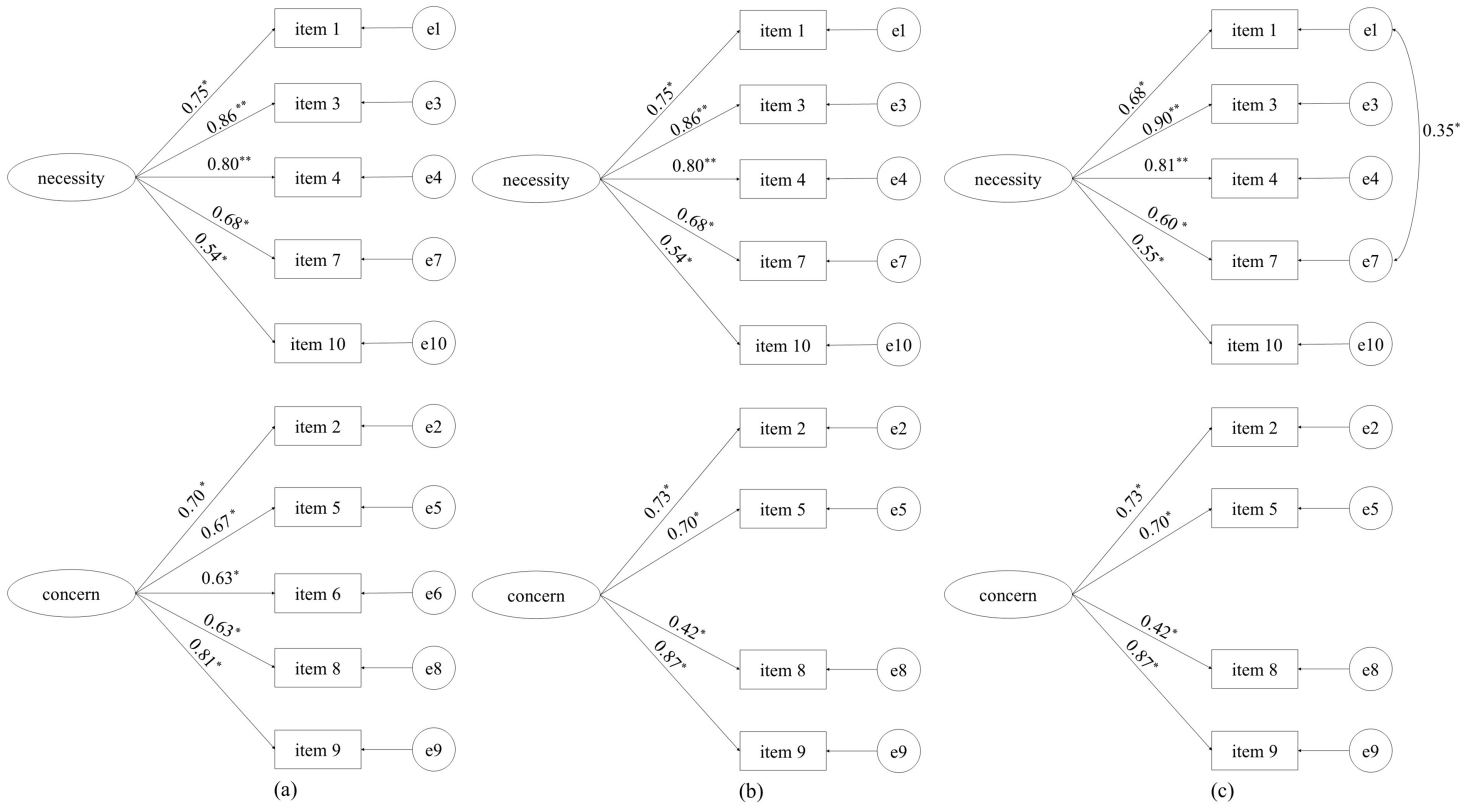
Confirmatory factor analysis of the Chinese Belief about Medicines Questionnaire (BMQ)–specific in caregivers of children with epilepsy (CWE). **(a)** Model 1, **(b)** Model 2, and **(c)** Model 3. * *p* < 0.05, **p < 0.01.

The initial CFA showed that Model 1 did not meet the cutoff criteria for goodness-of-fit. Studies have shown that the concept of “mystery” (item 6) is difficult to convey accurately during cross-cultural adaptation, so item 6 was deleted (Model 2) in this study. Finally, after modification based on the modification index—i.e., correlating error terms of items 1 and 7, which evaluate the same concept at different times—Model 3 showed significantly improved fit compared to Model 2 (∆*χ*^2^ = 241.867, ∆df = 1, *p* < 0.01), and the standardized factor loadings of all items were greater than 0.4 (Figure 1). Fit indices for all models are presented in Table 3.

**Table 3:** Fitness indices of three models.

Model	*χ* ^2^	df	CFI	TLI	SRMR	RMSEA (95% CI)
Model 1	1524.718	34	0.911	0.882	0.064	0.135 (0.129, 0.141)
Model 2	598.820	26	0.963	0.948	0.043	0.096 (0.089, 0.102)
Model 3	404.072	25	0.975	0.964	0.038	0.079 (0.073, 0.086)

CFI, comparative fit index; CI, confidence interval; df, degree of freedom; RMSEA, root mean square error of approximation; SRMR, standardized root mean residual; TLI, Tucker–Lewis index.

### 3.3 Results of the BMQ-S and MMAS-4

The results of the MMAS-4 indicated that 1,513 CWE (62.91%) were adherent to their medication therapy, and 892 (37.09%) were non-adherent. No significant differences were observed in the scores for the BMQ-N, BMQ-C, or the difference between the two scores when comparing adherence and non-adherence groups. Adherence rates across the four attitude groups showed no significant difference either (Table 4). In the unadjusted analysis, the BMQ-N score was not significantly associated with adherence (OR = 1.01, 95% CI: 0.89–1.15). This association remained non-significant after adjusting for covariates (OR = 1.07, 95% CI: 0.93–1.22). Similarly, analyses of the association between the BMQ-C and adherence yielded non-significant results, both before (OR = 0.95, 95% CI: 0.83–1.09) and after (OR = 0.92, 95% CI: 0.80–1.07) adjusting for covariates.

**Table 4:** Results of BMQ-S and MMAS-4.

Variables	Adherence	Non-adherence	Total	*χ*^2^ /*t* (*p*)
BMQ-N^#^	3.37 ± 0.65	3.37 ± 0.64	3.37 ± 0.65	0.18 (0.86)
BMQ-C^#^	3.66 ± 0.62	3.68 ± 0.59	3.67 ± 0.61	−0.72 (0.47)
Necessity concerns differential	−0.29 ± 0.89	−0.31 ± 0.81	−0.29 ± 0.86	0.64 (0.52)
Attitude				1.59 (0.66)
Indifference	330 (21.81)	193 (21.64)	523 (21.75)	
Acceptance	342 (22.60)	185 (20.74)	527 (21.91)	
Skepticism	364 (24.06)	215 (24.10)	579 (24.07)	
Ambivalence	477 (31.53)	299 (33.52)	776 (32.27)	

^#^The average scores for the BMQ-N and BMQ-C were calculated following the removal of item 6.

## 4 Discussion

To our knowledge, this is the first study to evaluate the psychometric properties of the Chinese BMQ-S among caregivers of CWE. Previous research has primarily focused on adults with epilepsy. Our findings support the use of the Chinese BMQ-S in this population.

The reliability analysis demonstrated acceptable reliability for BMQ-N and BMQ-C. These results are in line with previous research conducted in China, where Cronbach’s alpha values ranged from 0.60 to 0.92 for BMQ-N and from 0.58 to 0.91 for BMQ-C (21). Studies conducted in the United Kingdom (31), Sweden (16), and Turkey (32) have also reported similar outcomes. As such, the existing evidence suggests that the BMQ-S is a reliable tool across cultures.

The final model demonstrated good fit and supported the two-factor structure of the BMQ-S. The results were similar to those of Mervat et al. (33), who evaluated the psychometric properties of the Arabic BMQ-S in both children with chronic disease and their parents. Some studies seem to face similar challenges with the cultural adaptation of item 6 (“My medicines are a mystery to me”). For example, Maria et al. (16) first verified the reliability and validity of the Swedish BMQ in adults with epilepsy, ultimately changing the word “mystery” to “riddle” before formal analysis, although their results supported the original structure of the BMQ. Yang et al. also reported an issue of validity concerning item 6 compared to the original structure of the BMQ-S. These authors conducted principal component analysis and extracted items 6 and 8 together as a distinct dimension (23). All these studies indicate a problem accurately conveying the concept of “mystery” during cross-cultural adaptation, and the connotation of this term could potentially be altered and influence the validity of the BMQ. Furthermore, the AVE value of the original BMQ-C was less than 0.5, meaning that the five-item questionnaire explains less than 50% of the BMQ-C score—a finding consistent with the results of Beck et al. (34), although standardized factor loadings were greater than 0.4. The AVE value in this study increased to 0.54 after deleting item 6, suggesting that the Chinese item 6 may be unable to measure BMQ-C.

Of note, our RMSEA value was close to its threshold which may be associated with the degrees of freedom and the application of WLSMV in CFA. Kenny et al. pointed out that RMSEA does not have good performance with limited degrees of freedom (35). In addition, RMSEA thresholds were mainly established based on simulation studies using maximum likelihood estimation (36, 37). Recent studies have reported that RMSEA, CFI, and TLI all have problems when derived from diagonally weighted least squares models, whereas SRMR is considered a more reliable index (38, 39). In this study, the SRMR of Models 2 and 3 showed good fit. Thus, we conclude that the two-factor structure of BMQ-S is appropriate for use by caregivers of CWE.

The study found no significant relationship between BMQ-S scores and adherence. The rate of complete adherence observed in this study was higher than that reported in previous research, indicating potential overestimation of children’s adherence behavior by participants (40, 41). This report bias may obscure the actual relationship between BMQ-S and adherence, and studies conducted in America and Iran demonstrated similar results (41, 42). However, Mervat et al. identified both BMQ-N and BMQ-C scores as significant predictors of adherence (43). Mohamed N et al. reported the marginal positive association between non-adherence and the scores of BMQ-N (44). There are few consistent conclusions about the relationship between the BMQ and adherence among caregivers of CWE, indicating that further studies are needed.

### 4.1 Strengths and limitations

A key strength of this study is its large sample size. Furthermore, it addresses a gap in the literature by validating the BMQ-S among caregivers of CWE in China. However, several limitations should be noted. Due to its cross-sectional design, this study reflects the beliefs of caregivers at a single point in time within a specific region of Southwest China. As a result, its findings may have limited generalizability to other geographic contexts. Furthermore, while this study focused on caregivers, existing literature suggests there may be differences between caregivers’ beliefs and those of the children themselves. Future research should therefore explore the application of the BMQ-S directly in pediatric populations to assess their own perceptions of medicines.

## 5 Conclusion

This study is the first to validate the Chinese BMQ-S among caregivers of CWE, and it supports the notion that the BMQ-S is a reliable and valid instrument for assessing caregivers’ beliefs about antiseizure medicines. Further studies are needed to explore the relationship between the BMQ and adherence to medications.

## Data Availability

The raw data supporting the conclusions of this article will be made available by the authors, without undue reservation.
